# The complete mitochondrial genome of the Critically Endangered Angelshark, *Squatina squatina*

**DOI:** 10.1080/23802359.2017.1310609

**Published:** 2017-04-09

**Authors:** Cristín K. Fitzpatrick, Kimberly A. Finnegan, Filip Osaer, Krupskaya Narváez, Mahmood S. Shivji

**Affiliations:** a Save Our Seas Shark Research Center and Guy Harvey Research Institute, Nova Southeastern University, Dania Beach, FL, USA;; b Asociación Canaria para la Investigación y Conservación de los Elasmobranquios, ElasmoCan, Las Palmas de Gran Canaria, Spain;; c Fundación Colombiana para la Investigación y Conservación de Tiburones y Rayas, SQUALUS, Cali, Colombia

**Keywords:** Mitochondrial genome, *Squatina squatina*, Critically Endangered, Atlantic, Canary Islands

## Abstract

Here, we describe the first mitochondrial genome of the angelshark, *Squatina squatina.* The genome is 16,689 bp in length with 13 protein-coding genes, 22 tRNA genes, 2 rRNA genes, and a non-coding control region. Base composition of the mitogenome has an A + T bias (62.9%), seen commonly in other elasmobranchs. This genome provides a key resource for future investigations of the population genetic dynamics and evolution of this Critically Endangered shark.

The angelshark, *Squatina squatina* (Squatinidae), once ranged throughout the Northeast Atlantic, Mediterranean, and Black Sea. With a benthic lifestyle and low productivity, *S. squatina* is particularly Vulnerable to overexploitation, especially by demersal trawl fisheries (Miller 2016). Given estimates of over 80% depletion of its historical abundance, this species is designated as Critically Endangered on the International Union for the Conservation of Nature (IUCN) Red List (Ferretti et al. 2015). Despite its status as a species of high conservation concern, minimal genetic assessment of the angelshark has been performed to help inform conservation management.

Individual mitochondrial gene sequences are widely used to assess species diversity and population connectivity, however, whole mitogenome analysis can help identify regions of high variability, and provide higher resolution of intra-species diversity, connectivity, divergence time estimates, and phylogenies (Feutry et al. 2014). Here, we report the first mitochondrial genome sequence of the angelshark, *S. squatina.* The individual sequenced was caught as bycatch off the north east coast of Gran Canaria in the Canary Islands in 2008 as part of the islands’ artisanal fishery (Osaer et al. 2015). The sample (accession number OC-257) is stored in 100% ethanol at Nova Southeastern University, College of Natural Sciences and Oceanography.

Five overlapping sections of the *S. squatina* mitogenome were amplified by long PCR using five primer pairs designed from the consensus sequences of three published Squatinidae mitogenomes (*S. formosa* NC025328; *S. japonica* NC024276; *S. nebulosa* NC025578) with Geneious v.7.0.6 (http://geneious.com, Kearse et al. 2012) and its built-in Primer3 Design Tool (Untergasser et al. 2012). The PCR amplicons were pooled for library preparation with a Nextera XT DNA Sample Preparation kit (Illumina, San Diego, CA). Final whole mitogenome libraries were 2 × 250 bp paired-end sequenced, on an Illumina MiSeq sequencer. Reads were *de novo* assembled with CLC Genomics Workbench (QIAGEN) as well as aligned with reference-based assembler Bowtie2 (Langmead & Salzberg 2012) to resolve ambiguities. The mitogenome was annotated using MitoAnnotator (Iwasaki et al. 2013) and annotations confirmed by comparison to the three other published *Squatina* species.

**Figure 1.**
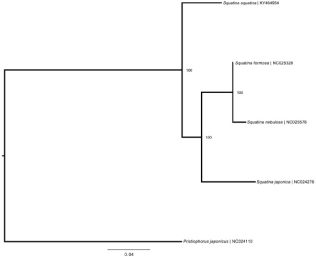
Bayesian tree depicting currently available Squatinidae mitogenomes with closely related outgroup. Scale and clade posterior probabilities are displayed. Labels include species name and GenBank RefSeq accession numbers.

The *S. squatina* mitogenome sequence (gb: KY464954) is 16,689 bp in length with a gene order typical of most vertebrates, with 13 protein-coding genes, 22 tRNA genes, 2 rRNA genes, and a non-coding control region (D-loop). Nucleotide composition leaned to an A + T bias with 30.9% A, 23.4% C, 13.7% G, and 32.0% T. The program MUSCLE was used to align the available squatinid mitogenome sequences on NCBI, with *Pristiophorus japonicus* (gb: NC024110) as an outgroup. A Bayesian phylogeny was constructed in MrBayes 3.2 (Huelsenbeck & Ronquist 2001; Ronquist & Huelsenbeck 2003) using the GTR + I as the best substitution model given by jModelTest v.2.1.10 (Darriba et al. 2012) Bayesian Information Criterion. The tree (Figure 1) shows the Atlantic restricted *S. squatina* as a sister species to the other three squatinids of Pacific origin. This first *S. squatina* mitogenome provides a genomic resource to aid conservation and management efforts for this highly depleted species.
